# Men’s involvement in family planning service utilization among married men in Kondala district, western Ethiopia: a community-based comparative cross-sectional study

**DOI:** 10.1186/s40834-021-00160-x

**Published:** 2021-06-01

**Authors:** Lemessa Assefa, Zemenu Shasho, Habtamu Kebebe Kasaye, Edao Tesa, Ebisa Turi, Ginenus Fekadu

**Affiliations:** 1 Department of Public health, Institute of Health Sciences, Wollega University, Nekemte, Ethiopia; 2 Deputy of the health center, Gimbi Health Center, Western Wollega Zone, Gimbi, Ethiopia; 3 Department of Midwifery, Institute of Health Sciences, Wollega University, Nekemte, Ethiopia; 4 Department of Public Health, College of Medical and Health Sciences, Madda Walabu University, Goba Referral Hospital, Goba, Ethiopia; 5 Department of Pharmacy, Institute of Health Sciences, Wollega University, P.O Box:395, Nekemte, Oromia Ethiopia; 6 School of Pharmacy, Faculty of Medicine, The Chinese University of Hong Kong, Shatin, New territory Hong Kong

**Keywords:** Men’s involvement, Family planning, Service utilization, Ethiopia

## Abstract

**Background:**

Men involvement is one of the important factors in family planning (FP) service utilization. Their limitation in the family planning program causes a decrease in service utilization as well as the discontinuation of the method which eventually leads to failure of the program. Family planning uptake is low but there is no enough study conducted on the parameters of husband involvement in Ethiopia. Hence, this study focused to assess men’s involvement in family planning service utilization in Kondala district, western Ethiopia.

**Methods:**

Community based comparative cross-sectional study design was employed in urban and rural kebeles of kondala district using quantitative and qualitative data collection tools. The multi-stage sampling method was employed to select 370 participants from each of the four urban and eight rural kebeles. Logistic regression analysis was used to identify variables that affect husbands’ involvement in FP service utilization. Statistical significance was declared at *p*-value of < 0.05 with 95% confidence interval (CI) and strength of association was reported by odds ratio (OR).

**Results:**

The study showed that 203(55.6%) men from urban and 178(48.8%) from rural were involved in FP service utilization. The median age of the respondents was 36+ 8.5 years (IQR: 27.5–44.5) in urban and 35 years (IQR: 25–45) in rural parts. Respondents who had four and above current children (AOR = 3.25, 95%CI = 1.51–7.02) in urban and (AOR = 4.20, 95%CI = 1.80–9.79) in rural were positively associated with men’s involvement in FP service utilization. In the urban setting, being government employee (AOR = 2.58**,** 95%CI = 1.25–5.33), wishing less than two children (AOR = 3.08, 95%CI = 1.80–5.24) and having a better attitude towards FP methods (AOR = 1.86, 95%CI = 1.16–2.99) were positively associated with FP service utilization. While good educational background (AOR = 2.13, 95%CI = 1.02–4.44), short distance from home to health facility (AOR = 2.29, 95%CI = 1.24–4.19) and having better knowledge (AOR = 4.49, 95%CI = 2.72–7.38) were positively associated with men involvement in FP service utilization in the rural area.

**Conclusion:**

Low involvement of men in family planning service utilization was reported in both settings. Factors associated with husbands’ involvement were varied between the two setups, except for the current number of children. Future FP program should incorporate infrastructure associated with the health facility, knowledge, and attitudinal factors.

## Background

Men involvement in family planning (FP) means that those participated by using family methods by themselves or made discussion with their wives on family planning, support and approve its utilization [1–5]. The most efficacious means of controlling population growth were pursued to turn the family planning reasoning on its head rather than looking to women’s adoption of family planning as the source of social change [6–8]. Traditionally the concept of reproductive health was more overemphasized on the females. However, from the day International Conference on population and Development (ICPD) made paradigm shift, men’s started to share responsibility and promote their active participation in liable parenthood, sexual and reproductive behavior including family planning [9, 10].

Family planning programs have paid attention primarily on women, though some studies suggest that involving males and obtaining their support and encouragement to family planning is of crucial importance, especially in developing countries where decision making are influenced by the men [7, 11–13].

Women are denied the use of contraceptives by her husband when tried to point out the involvement of the husband especially in decision making in reproductive health like in family planning [14, 15]. On the other hand, even if the wife wants to use family planning, she may not be able to use it or maybe forced to discontinue the method without the involvement of the males on the service [16]. A number of pocket research articles revealed that joint decision making and communications with partners in family planning issues will not only improve contraceptive utilization but also plays paramount role in averting discontinuation of the methods [14, 17, 18]. Men’s health status and behavior affect women’s health and reproductive health [6, 19]. Involving them increases their awareness, acceptance and support to their partners’ needs, choices and rights [6, 8]. Men’s involvement is also crucial to the success of family planning programs including the use of modern contraceptives [18–21]. They play this role by actively participating in family planning by using the method and discussion, decision making, support and approval of the service utilization by their wives, relatives and friends [7, 16, 22].

In many developing countries, several studies showed that men’s involvement brought a significant change in FP utilization. However, their involvement is still low in the patronage of family planning services [11, 15, 16, 23]. In Ethiopia, several studies have conducted in different parts of the country which concerned male involvement in reproductive health as well as in the utilization of family planning [4, 12, 14, 24, 25]. In this country, the husband has a great role in approving/disapproving the utilization of family planning services by their wives based on several barriers among these religions and cultures are stated to play negative influence on them [7, 24]. This is because in many developing countries like Ethiopia males often dominate in taking important decisions in the family including contraceptive use by their wives [18, 24].

Men as a decision-maker need to inform their wives to use FP and the discussion between the couples also play an important role in family planning. What was known by several studies as major factors to male involvement in family planning services utilization includes influence of culture and religion, perception towards family planning as women’s issue, lack of education of husband, sex preference for inheritance and fear of partner sexual promiscuity [19, 26, 27] . The presence of culture considers the husband as a decision-maker related to reproductive health, more specifically FP and it impeded the progress of service over the period despite the efforts being implemented to raise the utilization of FP services [19, 26]. These make low participation of the husband which has negative effects on the effectiveness of FP program as shown by studies conducted in several towns [19, 24, 27].

In Ethiopia even though the family planning uptake is low [28], most of the studies conducted on the parameters of male involvement and its determinants are conducted in the urban areas [29]. Little is known regarding male involvement in family planning utilization in the rural areas including the Kondala district. This study result creates an opportunity for couples to know about factors associated with husband involvement in family planning service utilization and can be used to revise the existing strategies and to design new ones to solve the problem associated with the program. Hence, the study aimed to explore the male involvement in family planning service utilization and associated factors in kondala district, Western Ethiopia.

## Methods

### Study area, period and design

Community based comparative cross-sectional study design was employed in urban and rural kebeles of kondala district from April to May 2019. Kondala district is found in the West Wollega zone, Oromia regional state, Ethiopia located 633 km away from Addis Ababa. The total population living in this district is about 1, 32,655 of whom 67,008 are males and 27,636 households. The district comprises a total of 36 kebeles, 32 rural and 4 urban kebeles. The major economic activities of the district are traditional farming and few of them are engaged in trading. The healthcare delivery within the district is carried out through 4 health centers, 33 health posts and 30 private clinics of different levels and one drug store. Family planning services are mainly obtained from public health institutions [30].

### Eligibility criteria

#### Inclusion criteria

- Men who were living together with their wives.
- Men who lived with their wives for at least in the past one year.
- Participants were expected to spend at least the past six months in the study area.

#### Exclusion criteria

- Men who were living in semi-urban kebele.

### Study variables

#### Dependent variable

Male involvement in family planning service utilization.

#### Independent variables

- Socio-demographic characteristics: Age, family size, religion, ethnicity, residence, educational status, occupation, and family income
- Reproductive health characteristics: Marriage duration, number of living children, knowledge regarding FP methods, the ideal number of children, attitude toward FP methods, health concern (fear of side effect, infertility, and low libido)

### Sample size determination and sampling techniques

The sample size was determined using a formula for estimation of double population proportion considering the following assumptions: level of significance (0.05), power (0.80), the proportion of husband who supports their wives to use contraceptive in urban (60%), and proportion of husband who supports their wives to use contraceptive in rural women (44.9%) [21]. The design effect and non-response rate were considered.

$$ \mathrm{n}={{\left({\mathrm{Z}}_{\upalpha /2}+{\mathrm{Z}}_{\upbeta}\right)}^2}_{\ast }\ \left({\mathrm{p}}_1{\left(1-{\mathrm{p}}_1\right)}_{+}\ {\mathrm{p}}_2\left(1-{\mathrm{p}}_2\right)\right)/{\left({\mathrm{p}}_1-{\mathrm{p}}_2\right)}^2; $$

Where**; −.**

Zα = the z-score corresponding to the 95% confidence level which is 1.96.

Z_β =_ is the critical value of the normal distribution at β which is 0.84

$$ \mathrm{n}=\frac{\left(\mathbf{1}.\mathbf{96}+\mathbf{0.84}\right)\mathbf{2}\ast \mathbf{0.60}\left(\mathbf{1}-\mathbf{0.060}\right)+\mathbf{0.449}\left(\mathbf{1}-\mathbf{0.449}\right)\Big)}{\left(\mathbf{0.60}-\mathbf{0.449}\right)\mathbf{2}.}=\mathbf{168} $$

By consideration of the design effect of 2 and the non-response rate of 10%, the total sample sizes were 370 from urban and 370 from rural kebeles.

The multi**-**stage sampling method was used in the sampling technique. Out of 32 rural kebeles, 8 kebeles were taken by simple random sampling and 4 urban kebeles were taken purposively. Households were sampled by a systematic random sampling method and the husband was asked from the household. The first households were selected by using the lottery method and the sample was taken every other K^th^ household until the required numbers of eligible husbands were recruited in the kebele. The K factor was derived from the formula K = N/n, where N is the total number of households in the kebele which were taken from district health office and n = sample size assigned for each kebele. Sample sizes for each rural and urban kebeles were allocated proportionally. If the husband has more than one partner/wife in one household, the husbands were asked for one partner taken by lottery method. In the case where the respondents were not found at the time of the study, a repeat visit was made for at least three times.

### Data collection process

Data was collected using quantitative and qualitative data collection tools. Quantitative data was collected using interviewer-administered structured questionnaires. The questionnaire was developed by the investigators after extensive review of literatures [19, 29, 31–33]. The questionnaire included socio-demographic characteristics, knowledge on family planning methods, the attitude of the husband towards family planning and husband involvement in family planning service utilization. Three health officers were assigned as research supervisors and six clinical nurses were trained and undertook the overall data collection activities under the immediate supervision of the principal investigator.

Focus group discussion (FGD) was conducted among purposively selected groups of key informants of the kebele to collect qualitative information on male involvement and factors associated with husband participation in family planning in the study area. The qualitative data was used to support the quantitative findings and it was carried out by the principal investigator. Three focus group discussions from urban kebeles and four from rural kebeles were conducted.

The groups were assigned according to their age; 18–39 years and 40 years or more. Each focus group comprised of 6 to 8 persons. The interviews and discussions were held in Afan Oromo language. The interview guide included questions on knowledge and attitude toward family planning, as well as factors towards male involvement in family planning. The principal investigator took data from FGDs by sound recording using mobile phone and notes.

### Data quality control and management

Questionnaires were translated to the regional language and then back translated to English to maintain its consistency. The training was given for data collectors and pretest was made on 5% of the study subjects in Gimbi district that have similar socio-demographic characteristics with the people of the study area before the actual data collection. The supervisors and principal investigators were performed immediate supervision daily. Every completed questionnaire was checked for its completeness and consistency.

### Measurement

#### Men involvement in family planning service utilization

Based on the summative score of questions designed to assess husband involvement in FP services utilization, husband those who score 60% and above were considered as having better involvement in FP services and those who score less than 60% were categorized as not involved [19].

#### Men’s attitude towards FP service utilization

To assess husband’s attitude towards involvement in F/P service utilization and they asked to answer five attitude related questions, which were a three points Likert’s scale. Based on the statements assessing attitude, the mean score of the distribution was considered as having positive attitude and those who score less than the mean value were categorized as negative attitudes towards family planning service utilization [19].

#### Knowledge about family planning methods

Refers to the husband’s knowledge of different family planning methods, which is based on knowledge question. Based on the summative score of questions designed to assess knowledge, men with above the mean of the distribution was considered as having adequate knowledge and those who score less than the mean value were categorized as not having adequate knowledge [19].

### Data processing, and analysis

The collected data were coded and entered into EPI info version 7.2.2.6 before the analysis. Then, data were exported and analyzed using Statistical Package for Social Sciences (SPSS) version 25. Mean and standard deviation as well as the median and interquartile range (IQR) was calculated for continuous variables, whereas frequency and proportion were calculated for categorical variables. The result was summarized and presented by statements, tables and graphs. Furthermore, binary logistic regression analysis was used to identify factors that affect husband involvement in family planning.

Variables having 0.25 and less *p*-value in bi-variable logistic regression model were candidates for multi-variable logistic regression model. The multi-variable logistic regression model was used to control the effect of confounding variables and the strength of association of predictor variables was assessed using odds ratio (OR) and significance of variables was reported at *p*-values < 0.05 using 95% CI. Model fitness was assessed by Hosmer and Lemeshow goodness of fit test (accepted with *p* > 0.05) and multicollinearity was assessed by variance inflation factor (VIF). Histogram and skewness were used for the normality check. Qualitative data were transcribed and back translated to English version, by senior experts, and the ideas rearranged according to their thematic area manually. Lastly, information linked and analyzed to its congruence with data obtained through interviewer-administered structured questionnaires.

## Results

### Socio-demographic characteristics of the study participants

A total of 365(98.6%) participants from urban and 364 (98.4%) from rural have participated in the study with a response rate of 729(98.5%). The median age of the respondents was 36 years (IQR: 27.5–44.5) in urban and 35 years (IQR: 25–45) in rural parts. Most husbands in urban 128(35.1%) attended secondary school and 120(33%) of rural areas attended primary school. Up on Chi-square (χ2) test, there is significant difference between urban and rural in terms of educational status, ethnicity, occupation and length of time spent to reach the family planning clinic (Table 1)

**Table 1:** Socio-demographic variables of husbands in urban and rural kebele of Kondala district, Western Wollega, Ethiopia, 2019

Socio-demographic variables		Urban (푛 = 365)	Rural (푛 =364)	p-value
Age (years)	20–30	78(21.4%)	98(26.9%)	0.14
	31–40	196(53.7%)	184(50.5%)	
	41–50	80(21.9%)	62(17%)	
	> 50	11(3%)	15(4.1%)	
Educational status	No formal education	55(15.1%)	120(33%)	< 0.001*
	Primary school	87(23.5%)	120(33%)	
	Secondary school	128(35.1%)	67(18.4%)	
	Diploma and above	95(26%)	57(15.7%)	
Ethnicity	Oromo	340(93.2%)	277(76.1%)	< 0.001*
	Amhara	11(3.0%)	9(2.47%)	
	Tigre	5 (1.37%)	4 (1.1%	
	Mao	9(2.5%)	74(20.3%)	
Religion	Protestant	74(20.3%)	41(11.3%)	0.001*
	Orthodox	71(19.5%)	58(15.9%)	
	Muslim	220(60.3%)	265(72.8%)	
Occupation	Merchant	106(29%)	28(7.7%)	< 0.001*
	Government employee	97(26.6%)	32(8.8%)	
	Farmer	93(25.5%)	287(78.8%)	
	Daily worker	69(18.9%)	17(4.7%)	
Length of time spent to reach the family planning clinic	< 30 min	340(93.2%)	112(30.8%)	< 0.001*
	30–60 min	25(6.8%)	137(37.6%)	
	> 60 min	–	115(31.6%)	

Note: *Statistically significant at p < 0.05

### Reproductive health characteristics of the respondents

Most of the respondents in both settings had duration of > 5 years of marriage [319(87.4%) in urban and 285(84.3%) in rural areas]. The median age at first marriage of the respondents was 24 (IQR: 18–30) among urban and 20 (IQR: 14–26) among rural participants. The mean (+SD) number of current children per husband was 3.44 + 2.2 in urban setting and 3.6 + 2.4 in rural settings (Table 2).

**Table 2:** Reproductive health characteristics of the study participants, in Urban and rural kebele of Kondala district, West Wollega, Ethiopia, 2019

Reproductive health characteristics		Urban, n (%)	Rural, n (%)	p-value
Duration of marriage (years)	0–5	46(12.6%)	79(21.7%)	0.002*
	> 5	319(87.4%)	285(78.3%)	
Age at first marriage	15–19	50(13.7%)	95(26.1%)	< 0.001*
	20–24	160(43.8%)	166(45.6%)	
	25–29	114(31.2%)	73(20.1%)	
	> 30	41(11.2%)	30(8.2%)	
Current number of children	None	60(16.4%)	58(15.9%)	0.81
	1–3	117(32.1%)	110(30.2%)	
	> 4	188(51.5%)	196(53.8%)	
Desired number of children	1–2	11(3%)	22(6%)	0.007*
	3–4	152(41.6%)	116(31.9%)	
	> 5	202(55.3%)	226(62.1%)	
Birth interval (years)	> 2	124(33.8%)	140(38.5%)	0.32
	3–4	123(33.7%)	105(28.8%)	
	3–4	83(22.7%)	76(20.9%)	
	> 4	35(9.6%)	43(11.8%)	

Note: *Statistically significant at p < 0.05

### Knowledge of husbands towards family planning methods

**Fig. 1:**
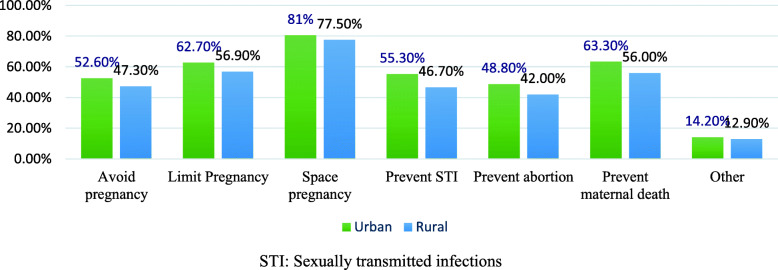
Knowledge of the study participants about the benefit of FP methods, in urban and rural kebele of Kondala district, West Wollega, Ethiopia, 2019

Overall, knowledge about family planning service utilization was 233(63.8%) in urban and 199(54.7%) in rural areas. In both settings, most of the husbands have ever heard about the family planning methods [361(98.9%) in urban and 352(96.7%) in rural areas]. Out of total study participants, 309(84.7%) in urban and 305(83.8%) in rural have heard about family planning methods from television and radio, respectively (Table 3). The benefit of family planning methods was mentioned mostly as birth spacing among 294(80.5% in urban and 282(77.5%) in rural participants (Fig. 1).

**Table 3:** Knowledge of study participant towards family planning in urban and rural kebele of Kondala district, West Wollega, western Ethiopia, 2019

Knowledge of study participant towards family planning		Urban, n (%)	Rural, n (%)
Ever heard about FP	Yes	361(98.9%)	352(96.7%)
	No	4(1.1%)	12(3.3%)
Source of information	Radio	212(58.1%)	305(83.8%)
	TV	309(84.7%)	130(35.7%)
	Health professional	267(73%)	280(76.9%)
	Neighbor	162(44.4%)	152(41.8%)
	Friends	194(53.2%)	176(47.4%)
	Wife	226(61.9%)	214(58.8%)
	Poster	205(56.2%)	191(58.8%)
	Other	54(14.8%)	22(6%)
Know side effect of FP	Yes	261(71.5%)	254(69.8%)
	No	100(27.4%)	98(26.9%)
side effect of FP experienced by their partner	Vomiting	108(29.6%)	95(26.9%)
	Abnormal menstrual	264(72.3%)	225(61.8%)
	Unwanted weight gain	152(41.6%)	139(38.2%)
	Headache	181(48.6%)	193(53%)
FP used by their partner	Pills	309(84.7%)	312(85.7%)
	Injectable	330(90.4%)	320(87.9%)
	Implant	255(69.9%)	236(64.8%)
	Loop	220(60.3%)	183(50.3%)
	Condom	119(32.6%)	99(27.2%)
	Calendar	136(37.6%)	127(34.9%)
	Permanent	197(54%)	167(45.9%)
	Breast feeding	208(57%)	178(48.9%)
FP for male	Condom	299(81.9%)	264(72.5%)
	Permanent	204(55.9%)	180(49.5%)
	Withdrawal	185(50.7%)	160(44%)
	Periodic Abstinence	189(51.8%)	156(42.9%)
	I don’t know	37(10.1%)	53(14.6%)
FP available for utilization	Pills	225(61.6%)	212(58.2%)
	Injectable	260(71.2%)	240(65.9%)
	Implant	146(40%)	120(33%)
	Loop	107(29.3%)	75(20.6%)
	Condom	211(57.8%)	196(53.8%)
	I don’t know	14(3.8%)	22(6%)
Where FP can be got	Hospital	107(28.9%)	87(23.9%)
	Health center	298(80.5%)	279(76.6%)
	Health post	145(39.2%)	161(44.2%)
	Private clinic	208(57%)	164(45.1%)
	Other	21(5.8%)	18(4.9%)
FP clinic arrangements special for male	Yes	177(48.5%)	162(44.5%)
	No	184(50.4%)	190(52.2%)
Any challenge to be involved in FP	Yes	194(53.2%)	190(52.2%)
	No	167(45.8%)	162(44.5%)
Challenges faced by Men’s to be involved in FP	Inadequate awareness of existing FP	34(9.3%)	63(17.3%)
	Religious prohibition	50(13.7%)	99(27.2%)
	Culture	58(15.9%)	83(22.8%)
	Shyness	70(19.2%)	123(33.8%)
	Negative community participation	74(20.3%)	129(35.4%)
	Lack of male FP service providers	31(8.5%)	60(16.5%)
	Fewer contraceptive choices for men	43(11.8%)	66(18.1%)

**Fig. 2:**
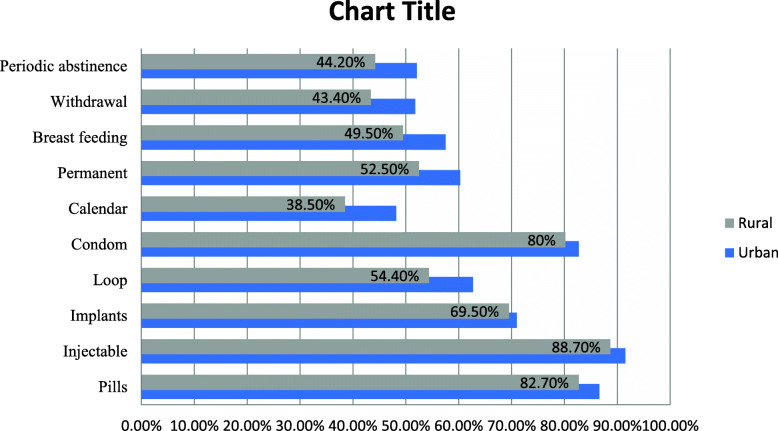
Most known FP among study participants, in urban and rural kebele of Kondala district, West Wollega, Oromia regional state, 2019

The most reported family planning method was injectable both in urban 334(91.5%), and rural area 323(88.7%) (Fig. 2)

**Fig. 3:**
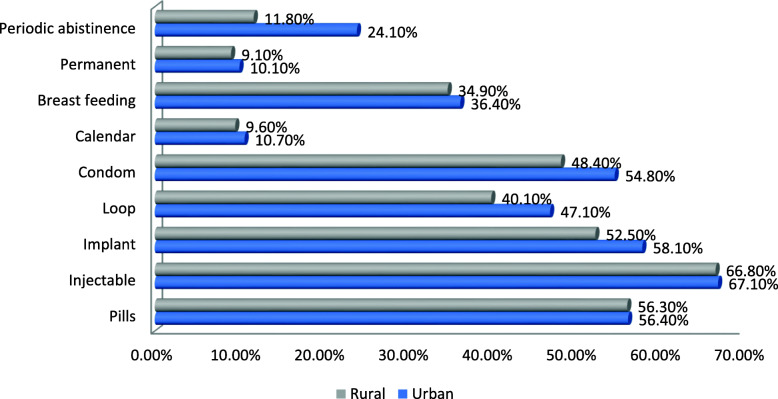
Safe method reported by participants, in urban and rural kebele of Kondala district, West Wollega, Oromia regional state, 2019

Most of the respondents [330(90.4%) in urban and 320(87.9%) in rural areas mentioned injectable as safest family planning method for females. About 299(81.9%) in urban and 264(72.5%) in rural area reported condoms as safest family planning method for men. Around 245(67.1%) of husband in urban and 243(66.8%) in rural area knew safe method of family planning as injectable **(**Fig. 3).

Family planning methods available for utilization identified during the study was injectable both in urban 260(71.2%) and rural areas 240(65.9%). According to their responses, most of the respondents were going to health center to use family planning, 295(80.8%) and 279(76.6%) in urban and rural, respectively. More than half of the participants in both rural and urban faced challenges to be engaged in family planning. About 74(20.3%) in urban and 129(35.4%) in rural of the respondents reported that negative community participation is among the most common challenges faced by the husbands to engage in family planning (Table 3).

The attitudes of husbands towards the involvement of family planning service utilization were assessed whether they had an interest to involve or not in the services. Husband’s opinions about their roles in FP service utilization were assessed on a five-tier scale of strongly agree, agree, neutral, disagree and strongly disagree. Accordingly, about half 188(51.5%) of respondents from urban disagreed that FP issue should concern only women and 135(37.1%) of husband from rural strongly disagreed. In FGD majority of the participants stated, *“By nature men cannot give birth; it is the women who give birth, so they should use family planning and not husband” (FGD community 1 from urban).* However; some participants raised *“It is not only women who face problems when the children lack what to eat, wear and materials for education so the family planning issue also concerns the men” (FGD community 3 from the urban).*

The study showed that about 159(43.6%) husbands from urban agreed that spouses can seek FP services without permission from husband but 117(32.1%) from rural strongly disagreed. About 158(43.3%) of husband from urban and 52(14.3%) from rural areas agreed that FP practice reduces confidence between husband and wife.

About 128(35.1%) of respondents from urban indicated neutral to discussion of FP issue with women as it is taboo and 133(36.5%) from rural strongly disagreed. About 91(24.9%) from urban strongly agreed that it is only women who are promiscuous that use FP without their husband consent and 72(31.6%) from rural indicated as they were strongly agreed. Generally, 204(55.9%) of urban and 102(28%) of rural husbands had a positive attitude towards contraceptive methods (Table 4).

**Table 4:** Attitude of husbands concerning family planning service utilization in urban and rural kebele of Kondala district, West Wollega, western Ethiopia, 2019

Attitude of husbands		Urban, n (%)	Rural, n (%)
FP issue should concern only women	Strongly agree	8(2.19%)	39(10.7%)
	Agree	26(7.1%)	43(11.8%)
	Neutral	59(16.2%)	52(14.3%)
	Disagree	188(51.5%)	83(22.8%)
	Strongly disagree	80(21.9%)	135(37.1%)
Spouse can seek FP services without permission from husband	Strongly agree	23(6.3%)	22(6%)
	Agree	159(43.6%)	52(14.3%)
	Neutral	19(5.2%)	77(21.2%)
	Disagree	92(25.2%)	84(23.1%)
	Strongly disagree	68(18.6%)	117(32.1)
FP practice reduces confidence b/n husband and wife	Strongly agree	39(10.7%)	26(7.1%)
	Agree	158(43.3%)	52(14.3%)
	Neutral	34(9.3%)	74(20.3%)
	Disagree	62(17%)	74(20.3%)
	Strongly disagree	68(18.6%)	126(34.6%)
Its taboo for men to discuss with women about FP	Strongly agree	28(7.7%)	22(6%)
	Agree	24(6.6%)	43(11.8%)
	Neutral	128(35.1%)	71(19.5%)
	Disagree	118(32.3%)	83(22.8%)
	Strongly disagree	63(17.3%)	133(36.5%)
It is only women who are promiscuous that use FP without their husband consent	Strongly agree	91(24.9%)	115(31.6%)
	Agree	53(14.5%)	39(10.7%)
	Neutral	86(23.6%)	72(19.8%)
	Disagree	88(24.1%)	67(18.4%)
	Strongly disagree	43(11.8%)	59(16.2%)
Only men need to decide on family planning	Strongly agree	54(14.8%)	25(6.9%)
	Agree	49(13.4%)	39(10.7%)
	Neutral	86(23.6%)	93(25.5%)
	Disagree	124(34%)	88(24.2%)
	Strongly disagree	48(13.2%)	107(29.4%)
FP methods decrease sexual urge	Strongly agree	46(12.6%)	29(8.0%)
	Agree	68(18.6%)	42(11.5%)
	Neutral	106(29%)	120(33%)
	Disagree	117(32.1%)	77(21.2%)
	Strongly disagree	24(6.6%)	84(23.1%)
Men should accompany their partners to FP clinics	Strongly agree	158(43.3%)	151(41.5%)
	Agree	159(43.6%)	66(18.1%)
	Neutral	14(3.8%)	31(8.5%)
	Disagree	16(4.4%)	57(15.7%)
	Strongly disagree	14(3.8%)	47(12.9%)
Husband involvement is important in FP	Strongly agree	158(43.3%)	113(31%)
	Agree	159(43.6%)	153(42%)
	Neutral	14(3.8%)	42(11.5%)
	Disagree	16(4.4%)	34(9.3%)
	Strongly disagree	14(3.8%)	10(2.7%)

### Involvement of husbands in family planning service utilization

The study revealed that out of study participants, 203(55.6%) with 95%CI (50.5–60.7) in urban and 178(48.9%) with 95%CI (44–54) in rural were involved in family planning service utilization by using or by encouraging their wives. About 135(37%) and 94(25.8%) of husbands in urban and rural have ever used condoms, respectively (Table 5**).** In FGD some participants stated that *“The perception of our community is not good and makes you not to use and support women on family planning usage, you perceived as you are jobless if you support your wife by accompanying her to health facility”(FGD community 4 from rural). “Using condom is not comfortable, I remember last two years I used after Dr. ordered me to use condom as not to transmitted to my wife when I become ill with sexual transmitted disease” (FGD community 2 from urban). “Using family planning is seen as acting opposite of Allah. Creator blessed human being as reproduce and fill the earth” (FGD community 4 from rural).* Similar responses were given in other group discussions.

**Table 5:** Involvement of husbands concerning family planning service utilization planning in urban and rural kebele of Kondala district, West Wollega, western Ethiopia, 2019

Involvement of husbands concerning family planning service utilization		Urban, n (%)	Rural, n (%)
Attended FP clinic	Yes	167(45.8%)	158(43.4%)
	No	194(53.2%)	200(53.3%)
How many times did you attended	Once	41(11.2%)	40(11%)
	Twice	54(14.8%)	51(14%)
	Many times	57(15.6%)	51(14%)
	I don’t remember	13(3.6%)	18(4.9%)
Method used by husband	Condom	135(37%)	94(25.8%)
	Permanent	6	7
Discussed with your wife on FP	Yes	272(74.5%)	212(58.2%)
	No	89(24.4%)	140(38.5%)
Frequency of discussion	Once	57(15.6%)	43(11.8%)
	Twice	44(12.1%)	45(12.4%)
	Many times	125(34.2%)	88(24.2%)
	I don’t remember	51(14%)	39(10.7%)
Initiated the discussion	Yes	214(58.6%)	136(37.4%)
	No	147(40.3%)	216(59.3%)
Approved FP for wife	Yes	212(58.1%)	144(39.6%)
	No	149(40.81%)	208(57.1%)
Reason for disapproval	Desire to have more child	105(28.8%)	107(29.4%)
	Fear of side effect	99(27.1%)	90(24.7%)
	Religious prohibition	89(24.4%)	73(20.1%)
	Fear of promiscuity	94(25.8%)	74(20.3%)
	Other	32(8.8%)	34(9.3%)
FP used by partner	Pills	214(58.6%)	177(48.6%)
	Injectable	216(59.2%)	182(50%)
	Implant	220(60.2%)	179(49.2%)
	Loop	201(55.1%)	176(48.4%)
	Permanent	39(10.7%)	34(9.3%)
	Calendar	198(54.2%)	176(48.4%)
	Breast feeding	190(51.1%)	169(46.4%)
Reason for utilization	For birth spacing	196(53.7%)	138(37.9%)
	For birth limiting	27(7.4%)	42(11.5%)

According to participants responses, 272(74.5%) from urban and 212(58.2%) from rural married men discussed family planning with their partner in the last 1 year. Of those who discussed about family planning 214(58.6%) from urban and 136(37.4%) from rural husbands were initiated the discussion on family planning issues (Table 5). In FGD some participants stated; *“The current situation in terms of economic hardship cannot be explained and this factor ordered you to limit the number of children this was why we ordered to discuss with our wife and told her she had used family planning so that we can take good care for ourselves and our children”(FGD Community 2 from Urban). “Allah who gives you a child will not ignore you on their growth no need of discussion on this matter” (FGD community 3 from rural). “Sometimes children can be counted as an asset; they will support you when you become old. There was a person who die without any care and buried without respect like other animals because he has no child, therefore the discussion that inhibits childbirth is not goods” (FGD Community 4 from rural).*

About 212(58.1%) and 144(39.6%) of the husbands from the urban and rural, respectively approved the use of family planning methods (both the male and female method). The main reasons for disproval were desire to have more children in both urban 105(28.8%) and rural 107(29.4%) parts of the respondents (Table 5). In qualitative part (FGD) participants stated that; − *“The type of our occupation makes us have more children; let God show you if the children limited to 3 or 4 who can act as housekeeper, shepherd, who should help the father at farming place and who should help the mother at home this is why family planning usage can be disapproved” (FGD community 1 from Rural). “Ahh …*. *Women cannot be obeyed if they not caught at least by two to three years with birth-giving” (FGD community 3 from rural).*

About 222 (60.8%) from urban and 178(48.9%) from rural husbands’ partners were used family planning and the most preferred contraceptive method in urban was implant 220(60.3%) and injectable 182(50%) in rural area. The main reason reported for utilization of family planning method was for child spacing in both setting 196(53.7%) and 138(37.9%) in urban and rural respectively. The study also revealed that around 266(72.9%) of urban and 193(53%) of rural husbands have supported their wives. About 257(70.4%) of husbands in urban supported by accompanying their wives to health facility and 190(52.2%) from rural supported by financial. About 67.7% from urban and 52.2% from the rural support their wives psychologically.

In FGD, most participants they stated that;-*“In the past 3 three years my wife bleeds profusely on delivery and it was only God intervention that safe my wife after that we have decided to use family planning and my support was never stopped starting from that occasion even I am going with her the health center” (FGD community 2 from rural)*. *“It is good to support partners in family planning usage because the women can forget the appointment and unwanted childbearing might have happened” (FGD community 1 from urban)*. Other participants did not see its importance and they stated, *“It feels you shy to accompany women for family planning services to health facility. It is good if men with men and women with women” (FGD community 2 from rural). “There is no favorable option for husbands like women to use FP so how men can support his partner so she should uses as given for her from God” (FGD community 3 from urban).*

About 263(72.1%) in urban and 227(62.4%) in rural respondents were discussed with their friends on family planning issues. About 251(68.8%) from urban and 221(60.7%) from rural encouraged their friends in family planning service utilization. Out of the respondents about half [203(55.6%) from urban and 168(46.2%) from rural] had participated in community mobilization in family planning service utilization issues. During the group discussion, some participants stated that; *“As you know there is no method of choice for men if you tied yourself you will not able to have sexual intercourse again and with condom, you cannot enjoy sex so I have no reason to encourage my friend to family planning” (FGD community 1 from urban). “We had accessed to similar source of information, but I have encouraged my friends who had many children because he discontinued his wife FP usage fearing its side effect” (FGD community 1 from urban).* Similar suggestions were obtained from the rest FGD discussions

### Factors that influence husband involvement in FP service utilization

#### Bi-variable logistic regression analysis with selected variables

On bi-variable logistic regression, out of fifteen independent variables, six variables from urban and five variables from rural had an association with the outcome variable at *p*-value of 0.25. Based up on this husbands with educational level of diploma and above were nearly three times more likely to be involved in FP utilization in rural (COR = 2.89; 95% CI: 1.46–5.73) and urban COR = 2.42; 95%CI 1.19, 4.88) respectively. Regarding occupational status, government employee are about two times (COR = 2.07(1.09, 3.90) more likely to be involved in FP utilization in urban. Husbands who walk less than 30 min from health facility are about 2.29 times more likely to be involved in FP utilization than those who walkt for > 60 min (COR = 2.29; 95% CI: 1.34,3.92) in the rural. Those husbands who have been together for morethan five years since married are about three times (COR = 3.29, 95%CI: 1.68–6.44) in urban and about 1.7 times (COR = 17; 95%CI: 1.02–2.85) in rural to be involved in the FP utilization. Those couples who have four and above children are about four times (COR = 4.69; 95%CI: 2.4–9.16) in rural and about three times (COR = 3.50; 95%CI:1.89–6.47) in urban to be involved in FP. For urban husbands, those who have desire to have 3–4 children are about two times (COR = 2.12;95%CI: 1.37–3.29) more likely to be involved in FP utilization. For rural respondents, those who have adequate knowledge are nearly five times (COR = 4.58, 95%CI: 2.91,7.21) more likely to be involved in FP utilization than those who have inadequate knowledge. For urban husbands, those who have positive attitude towards FP service utilization are about two times 2.02(1.32,3.08) more likely to be involved in FP utilization (Table 6).

**Table 6:** Bi-variable analysis of factors contributing to husbands’ involvement in family planning service utilization in urban and rural kebele of Kondala district, West Wollega, western Ethiopia, 2019

Variable	Category	Husband Involvement in FP				OR (95%CI)	
		Urban		Rural		Urban	Rural
		Involved (%)	Not involved (%)	Involved (%)	Not involved (%)		
Educational status	No Formal education	29(53.7)	2546.29)	54(46.95)	61(53.04)	1	1
	Primary school	41(48.23)	44(51.76)	51(44.35)	64(55.65)	0.80(0.40, 1.59)	0.90(0.54, 1.51)
	Secondary school	63(49.6)	64(50.3)	32(49.23)	33(50.76)	0.85(0.45, 1.60)	1.09(0.59, 2.01)
	Diploma & above	70(73.68)	25(26.3)	41(71.92)	16(28.07)	2.42(1.19, 4.88) *	2.89(1.46, 5.73) *
Occupational status	Merchant	58(55.76)	46(44.23)	**	**	1.23(0.66, 2.25)	**
	Government employee	66(68.04)	31(31.95)			2.07(1.09, 3.90) *	
	Farmer	44(48.35)	47(51.64)			0.90(0.48, 1.70)	
	Daily Worker	35(50.72)	34(49.27)			1	
Distance to HF (min)	< 30	**	**	66(61.11)	42(38.89)	**	2.29(1.34,3.92)*
	30–60			66(50.38)	65(49.61)		1.48(0.89,2.45)
	> 60			46(40.7)	67(59.29)		
Duration of marriage (years)	0–5	14(31.11)	31(68.88)	31(40.25)	46(59.74)	1	1
	> 5	189(59.81)	127(40.18)	147(53.45)	128(46.54)	3.29(1.68,6.44)*	1.70(1.02,2.85)*
Current number of children	None	21(35.59)	38(64.40)	14(24.13)	44(75.86)	1	1
	1–3	60(51.28)	57(48.71)	52(48.59)	55(51.41)	1.90(1.00,3.63)*	2.97(1.46,6.05)*
	> 4	122(65.94)	63(34.06)	112(59.89)	75(40.11)	3.50(1.89,6.47)*	4.69(2.40,9.16)*
Desired number of children	1–2	4(36.36)	7(63.63)	**	**	0.59(0.16,2.09)	**
	3–4	102(67.10)	50(32.89)			2.12(1.37,3.29)*	
	> 5	97(48.89)	101(51.01)			1	
Knowledge about FP	Not Knowledgeable	**	**	46(30.06)	107(69.93)	**	1
	Knowledgeable			132(66.33)	67(33.66)		4.58(2.91,7.21)*
Attitude Towards FP	Negative attitude	73(46.49)	84(53.51)	**	**	1	**
	Positive attitude	130(63.72)	74(36.27)			2.02(1.32,3.08)*	

* Variables which are significant at 95%CI with p-value < 0.05, ** Data which are not included in the model, 1: reference category

*COR* Crudes Odds Ratio; *FP* Family planning; *HF* Health facility

#### Multi-variable logistic analysis of husband involvement with associated factors

Out of six from urban and five from rural variables which were candidate for multi-variable logistic regression analysis, 4 variables both in urban and rural were statistically significant with the husbands’ involvement in FP service utilization at *p* < 0.05. In Urban areas, husbands who participated in government employees were about 2.5 times more likely to be involved in family planning service utilization than other types of occupational status (AOR = 2.58,95%CI = 1.25–5.33, *p* = 0.01). Respondents who had four and above or a greater number of current children were 3.25 times more likely to be involved in family planning service utilization than those who had less than four children. (AOR = 3.25, 95%CI = 1.51–7.02, *p* = 0.003). Husbands who had desired number of children less than two were 3 times more likely to be involved in family planning service utilization than husbands who had desired number of children more than two (AOR = 3.07, 95%CI = 1.80–5.24, *p* = 0.003. Finally, husbands who had positive attitude towards family planning methods were 1.86 times more likely to be involved than those who had negative attitude in family planning service utilization (AOR = 1.86, 95%CI = 1.15–2.99. p = 0.01).

In rural setting, husbands who had diploma and above were about two times more likely to be involved in family planning service utilization than those who had below educational background (AOR = 2.13, 95%CI = 1.02–4.44, *p* = 0.05). Husband who goes less than 30 min to reach health facility to get service were 2.28 times more likely to be involved in family planning service utilization than those who traveled more than 30 min (AOR = 2.28, 95%CI = 1.24–4.19, *p* = 0.008). Participants who had four and above number of current children were about 4 times more likely to be involved in family planning service utilization than those who had less than four children. (AOR = 4.27, 95%CI = 1.88, 9.73, *p* = 0.001). Lastly, husbands who were knowledgeable to family planning methods were 4.48 times more likely to be involved than husbands who were not knowledgeable in family planning service utilization (AOR = 4.48,95%CI = 2.72–7.38, *p* < 0.001) (Table 7)

**Table 7:** Multi-variable logistic analysis of husband involvement in family planning service utilization with associated factors in urban and rural kebele of Kondala district, West Wollega, western Ethiopia, 2019

Variable	Category	Urban areas		Rural areas	
		AOR (95%CI)	p-value	AOR (95%CI)	p-value
Educational status	No Formal education	1	1	1	1
	Primary school	0.851(0.39, 1.82)	0.676	0.928(0.52, 1.66)	0.802
	Secondary school	0.806(0.39, 1.65)	0.556	1.198(0.60, 2.38)	0.606
	Diploma and above	2.193(0.99, 4.83)	0.051	2.126(1.02, 4.44)	0.045*
Occupational status	Merchant	1.378(0.70, 2.69)	0.349	**	**
	Government employee	2.585(1.25, 5.33)	0.010*		
	Farmer	0.828(0.41, 1.66)	0.595		
	Daily Worker	1	1		
Distance from home to HF	< 30 min	**	**	2.283(1.24, 4.19)	0.008*
	30–60 min			1.251(0.70, 2.23)	0.447
	> 60 min			1	1
Duration of marriage	0–5 years	1	1	1	1
	> 5 years	2.114(0.94, 4.75)	0.070	1.088(0.54, 2.18)	0.812
Current number of children	None	1	1	1	1
	1–3	1.304(0.61, 2.78)	0.493	4.275(1.88, 9.73)	0.001
	> 4	3.255(1.51, 7.02)	0.003*	4.204(1.80, 9.79)	0.001*
Desired number of children	1–2	2.217(0.55, 8.93)	0.263*	**	**
	3–4	3.076(1.80, 5.24)	< 0.001*		
	> 5	1	1	1	1
Knowledge about FP	Not Knowledgeable	**	**	1	1
	Knowledgeable			4.478(2.72, 7.38)	< 0.001*
Attitude Towards FP	Negative attitude	1	1	**	**
	Positive attitude	1.863(1.15, 2.99)	0.010*		

* Variables which are significant at 95% CI with p-value of < 0.05, ** Data which are not included in the model, 1 reference category

*AOR* Adjusted odds ratio; *HF* Health facility

## Discussion

The finding of the study revealed that more than half (55.6%) from urban and (44.9%) from rural husbands were involved in family planning service utilization in the district. This was low when compared with world health organization (WHO) attention towards male involvement in reproductive health including family planning service utilization [34]. The reasons for this low finding might be due to low provision of information on service utilization, low service targeting men within the health facilities and low attention to satisfied users to promote the usage of the methods. The involvement in service utilization was higher in urban compared to the rural areas. This might be because in rural areas negative community perception on husbands supporting ladies, health facility setup and the distance from home to health facility negatively influenced husbands’ involvement in family planning service utilization. However, the service utilization was higher than the study results in west Shoa (36%) [27] and in India 22% in urban and 17% in rural [35]. The discrepancy may be due to the improvement of the health facilities on the provision of the family planning service with time variation.

However, the current study on husbands’ involvement in family planning service utilization was lower than similar studies conducted in Gedeo zone, (70.9%) in urban and (63.4%) in rural areas [21], Agaro town 74.4%) [7], Asosa (70%) [26], urban part of Nigeria (80%) [11] and in rural areas of India (West Bengal) (74.4%) [36]. This discrepancy perhaps be due to most of the indicated studies are conducted in the urban areas [7, 26] where majority of them are supposed to be literate and have access to information and services.

This study also attempted to identify factors associated with husband involvement in family planning service utilization. Socio-economic status, husband’s reproductive characteristics, Knowledge and attitudes towards FP service utilization were among factors associated with the involvement of males in FP services utilization.

It revealed that there is positive association of husband’s FP knowledge with the involvement in FP service utilization, especially in rural parts. The finding complements to other studies done in Tigray, Debremarkos, Gedeo zone, Bahir Dar and Afar [4, 5, 14, 19, 21]. This is because knowledge is pre-request to be involved in family planning service utilization. Additionally, husband’s having positive attitude towards FP were 1.86 times more likely to be involved family planning service utilization than those having negative attitude in urban setting. This is supported by the previous study conducted in Nigeria, India and Gedeo zone [21, 35, 37].

In Urban areas, husbands who were government employees were nearly three times more likely to be involved in family planning service utilization than other types of occupational status. This finding is supported by similar studies conducted in Ethiopia; Angecha woreda [12], Bahirdar town [29]. This is due to the fact that most of government employees in Ethiopia are educated and this in turn associated with the statement that the more a husband is educated, the more he will accept gender equality and believe in equal participation in decision making [38].

This indicated that the more educated respondents are more familiar with family planning methods and utilizes it to give special care for their having children. Respondents who had a greater number of current children were about 3 times in urban and 4 times in rural more likely to be involved in family planning service utilization compared to individuals who had no more children. This finding was in line with the studies conducted in Asosa town, North Shoa and Loka Abay district [18, 20, 26]. However, the finding was contrary to study conducted in urban area (Bahir Dar) where involvement in family planning was higher among individuals who had no or less children than who had more children [14]. The reason for the discrepancy in the current study might be because the current living expense was becoming difficult to have more children related to the current economic hardship in the country.

From this study husbands who do not want to have any more children were more likely to be involved in family planning service utilization than husbands who want to have more children. This finding was supported by the study conducted in Kembata Tembaro zone [12]. However, educational status and duration of marriage did not show association with husband involvement in family planning service after confounding was controlled in urban setting.

According to the findings of this study educational status was one significant predictor of husbands’ involvement on family planning in rural areas. It showed as the educational status of respondents increases, there showed an evidence of husbands’ involvement in family planning service utilization. This was supported by previous studies conducted in different areas and countries where education was significantly affected family planning service utilization [14, 18, 25, 39]. The study also revealed that husband who goes less than 30 min from home to health facility to get service were about 2 times more likely to be involved in family planning service utilization than those who traveled more than 30 min. However, duration of marriage toward family planning did not show strong association with husband involvement following controlling confounder in rural areas.

The study assessed through qualitative way also identified husbands’ perception towards involvement in family planning service utilization. Most of the husbands in urban area perceived that they believe in the involvement of males in FP issue as it was not the only issue of the women. In rural areas the participants’ perception was not far from the answer to quantitative questions. Culturally perceived reproductive issues as the women business, desire of more children, fear of side effects of family planning and fear of community perception determined them as not to be involved as much like in urban areas. Generally, husbands living in urban areas were more likely to be involved than their rural counterparts. This finding was in line with the study conducted in Kembata Tembaro zone and India [12, 35]. The discrepancy could be due to in urban areas relatively good income, easily access to health facilities, educational facilities, broadcasting facilities and other services are more accessible than rural counterpart.

As strength, qualitative method was used to supplement the result and to explore factors that were not addressed by quantitative survey. Our study is not without some limitations. The result of this study totally relied on self-report and willingness of the participants to give real information and thus does not provide an objective measure of husbands’ involvement in family planning.

## Conclusion

The finding of the study showed the proportion of husbands’ involvement in family planning utilization in urban and rural areas was low. Factors associated with the involvement varied between the two setups, except the current number of children. Being government employee, wishing less than two children and having a better attitude towards family planning methods in urban and good educational background, less distance from home to health facility and having better knowledge were factors associated with husband involvement in family planning utilization in rural areas. In the qualitative study, most husbands in rural areas were not experienced good perception like urban areas in the involvement of FP service utilization due to different Socio-cultural factors.

Hence the governmental (the district and zonal health office) and non-governmental organizations working on the family planning issues should address socio-cultural, infrastructure associated with health facility, knowledge and attitudinal factors in future family planning programs. Besides family planning service utilization interventions in the area need to be promoted, awareness creation should be made, and health extension worker should enhance health education especially in rural part in family planning service utilization.

## Data Availability

The datasets used and/or analyzed during the current study are available from the corresponding author on reasonable request.

## Abbreviations

AOR: Adjusted Odd Ratio
CI: Confidence Interval
FGD: Focused Group Discussion
FP: Family Planning
IQR: Inter Quartile Range
IUCD: Intra Uterine Contraceptive Device
MOH: Ministry of Health
NGO: Non-Governmental Organizations
OC: Oral Contraceptive
OR: Odds Ratios
RH: Reproductive Health
SPSSS: Statistical Package for Social Sciences
WHO: World Health Organization
